# Beyond playing games: nephrologist vs machine in pediatric dialysis prescribing

**DOI:** 10.1007/s00467-018-4021-4

**Published:** 2018-07-12

**Authors:** Wesley Hayes, Marco Allinovi

**Affiliations:** 1 Great Ormond Street Hospital, London, UK; 2 University College London Institute of Child Health, London, UK; 3 Meyer Children’s Hospital, Florence, Italy

**Keywords:** Artificial intelligence, Machine learning, Renal dialysis, Body water, Child

## Abstract

In a recent article in Pediatric Nephrology, Olivier Niel and colleagues applied an artificial intelligence algorithm to a clinical problem that continues to challenge experienced pediatric nephrologists: optimizing the target weight of children on dialysis. They compared blood pressure, antihypertensive medication and intradialytic symptoms in children whose target weight was prescribed firstly by a nephrologist, then subsequently using a machine learning algorithm. Improvements in all outcome measures are reported. Their innovative approach to tackling this important clinical problem appears promising. In this editorial, we discuss the strengths and weaknesses of their study and consider to what extent machine learning strategies are suited to optimizing pediatric dialysis outcomes.

## Introduction

The capabilities of artificial intelligence (AI) and machine learning have advanced considerably in recent years. Consistent proficiency in highly complex tasks is now feasible, as showcased by a team at Google DeepMind who developed a self-taught algorithm that mastered the ancient Chinese board game Go, a feat that was previously only considered possible by the human brain [1].

The application of AI and machine learning in healthcare is rapidly expanding. A pilot study suggested AI may be beneficial for clinical decision support in anemia management [2], and a further clinical trial is underway [3]. Neural network models were found to outperform pathologists in classification of kidney biopsy samples [4]. Use of machine learning to predict acute kidney injury and chronic kidney disease from large and complex patient data streams has shown promising results [5].

One of the key challenges in pediatric dialysis care is optimization of children’s target weight. This is currently estimated by experienced nephrologists with variable use of objective measures [6]. In this edition of Pediatric Nephrology, the study by Niel et al. applied a machine learning algorithm to this problem [7].

## The challenge of optimizing target weight in pediatric dialysis patients

Determining target weight is the most challenging aspect of the pediatric dialysis prescription, compounded by the lack of a gold standard measure of fluid overload. This is an important clinical problem to address, because chronic fluid overload is a major risk factor for cardiovascular sequelae [8] and, ultimately, mortality for children with end-stage kidney disease [9]. Conversely, excessive ultrafiltration rates cause myocardial stunning and hasten decline in residual kidney function [10]. Optimizing target weight in pediatric dialysis prescriptions is therefore paramount.

Clinical assessment of target weight is notoriously unreliable. Fluid overload is not detectable by physical examination until levels approach 10% bodyweight. Target weight changes continuously in children due to nutritional growth [6].

A number of objective measurements to aid assessment of target weight have been developed, including bioimpedance spectroscopy [11], inferior vena cava collapsibility index [12], relative blood volume monitoring [13], N-terminal pro-brain natriuretic peptide [14] and lung ultrasound [15]. In addition, further data from ambulatory blood pressure monitoring, intradialytic trends in cardiac output, heart rate and core-peripheral temperature gap may enhance the assessment. Whilst individual measures can improve target weight estimation when used alone, each measure has different strengths and weaknesses. A multi-parameter assessment is therefore desirable. To date, successful assimilation and analysis of these multiple data streams in order to optimize target weight has not been achieved.

## Artificial intelligence is suited to optimizing target weight

AI machine learning techniques are well placed to address the challenge of processing multiple data streams from children on dialysis to optimize their target weight for a number of reasons:

Firstly, AI approaches differ from classical statistical techniques in their lack of reliance on assumptions about the underlying data. For example, a classical linear regression model assumes a linear relationship between dependent and predictor variables, no multi-collinearity and at least 20 cases per predictor variable. None of these criteria would be fulfilled by all of the measures of fluid overload discussed above. Classical statistical models are therefore not well suited to a multi-parameter assessment of target weight. Conversely, AI techniques algorithmically represent data structure to make predictions and classifications with fewer underlying assumptions and are therefore better suited to a multi-parameter analysis of fluid measures.

A further advantage of AI is the capability to tease out complex associations which cannot easily be reduced to an equation. Patterns representing fluid overload from the measures discussed above are inherently complex, hence the current reliance on experienced nephrologists to assess trends. Machine learning techniques can learn from large volumes of data, vastly exceeding the number of cases experienced by an expert clinician in a lifetime. Neural networks are one form of machine learning; data are processed by layers of “neurons” which take variable combinations of output from previous layers to generate new output thus mimicking neuronal processing in the brain, thereby facilitating highly complex data analysis.

## Results of the study by Olivier Niel et al.

Olivier Niel et al. applied a multi-layer neural network to optimize target weight in a cohort study of 14 children on hemodialysis [7]. They compared blood pressure, antihypertensive medication and intradialytic symptoms in children whose target weight was prescribed firstly by a nephrologist, then by a neural network algorithm. Improvements in all outcome measures are reported including reduction in median post-dialysis blood pressure from 77th to 60th centile, reduction in antihypertensive medication in 4 of 14 cases and reduction in intradialytic symptoms in three patients. Their innovative approach to tackling an important clinical problem which continues to challenge experienced pediatric nephrologists is commendable.

In order to understand if this study provides a generalizable solution to optimizing target weight, we will consider its limitations. The primary limitation was the small sample size, which is common to all single-center pediatric dialysis studies. The cohort design without blinding was vulnerable to bias, in particular in outcomes reporting intra-dialytic symptoms and reduction in antihypertensive medications. Exclusion of small patients weighing less than 20 kg limits applicability because target weight in infants is more challenging to assess. Evaluation of longitudinal cardiovascular outcome measures such as carotid intimal medial thickness and left ventricular hypertrophy, and loss of residual kidney function, would have been desirable.

Given the limitations of this first pilot study applying machine learning to optimize target weight, the results are not sufficiently robust to recommend use of this strategy in clinical practice at this point in time. This is however an important study which illustrates the potential of neural networks to improve pediatric dialysis outcomes and should trigger further work to progress this approach.

## Future work

The neural network algorithm used by Neil et al. used data from three sources, namely, bioimpedance spectroscopy, relative blood volume monitoring and post-dialytic blood pressures. The power of a machine learning solution lies in the capacity to synthesize large volumes of data from multiple inputs and may not be fully utilized with a three-input model. In order to exploit the capabilities of machine learning, further data could be analyzed such as lung ultrasound, N-terminal pro-brain natriuretic peptide, indexed inferior vena cava diameters and continuous data streams from ambulatory blood pressure monitoring.

A broad range of machine learning techniques have been developed which require differing approaches to network training, ranging from supervised to unsupervised strategies facilitated by the development of “deep learning” in recent years [16]. Neil and colleagues evaluated the performance of a single machine learning technique; collaboration between clinical and AI teams is called for in order to find the most powerful analysis strategy in future studies.

The ultimate goals in optimizing target weight in children on dialysis are to improve their cardiovascular health and preserve residual kidney function. Outcomes which reflect these goals such as carotid intimal medial thickness [17], echocardiographic assessment of ventricular mass and myocardial dynamics [18] and change in residual kidney function should be evaluated in future studies.

## Conclusions

The pilot study by Neil et al. has introduced a novel approach to tackling the longstanding clinical challenge of optimizing target weight in the pediatric dialysis prescription with encouraging initial results [7]. The capacity of machine learning algorithms to synthesize large volumes of data from multiple sources to undertake complex tasks is now well established, and their application to pediatric dialysis has vast potential. Further studies evaluating a range of machine learning strategies to process multiple data sources in order to improve cardiovascular markers in children on dialysis are merited.
